# Vaccines for Women Age 50 and Older^1^

**DOI:** 10.3201/eid1011.040469

**Published:** 2004-11

**Authors:** Pierce Gardner, Sudha Pabbatireddy

**Affiliations:** * National Institutes of Health, Bethesda, Maryland, USA;; † Stony Brook University, Stony Brook, New York, USA

**Keywords:** Immunization recommendations, older populations, women, immunity, travel, conference report

## Abstract

For older populations, most of whom are women, preventing illnesses and deaths through the use of vaccines is a leading public health challenge. Our understanding about how age and sex affect the immune system is limited, and basic and translational research aimed at improving vaccines and immune responses of older persons is needed. In the meantime, fully implementing current vaccine recommendations, particularly those for influenza and pneumococcal vaccines, can save thousands of lives and prevent illnesses in persons >50 years of age.

## Abstract 2

Implementing current vaccine recommendations can save thousands of lives and decrease illness among persons 50 years of age and older.

In the United States, the elderly are more likely to die of a vaccine-preventable disease. Adult deaths from influenza (≈36,000/y) (*1**,**2*), invasive pneumococcal disease (≈9,000/y) (*3*), and hepatitis B (≈5,000/y) (*4*) exceed vaccine-preventable deaths among children (≈50/y) by a ratio of ≈1,000:1. For each of these diseases, case-fatality rates rise with increasing age. This disparity can be addressed through adult vaccination programs, which are cost-effective and life-saving. Women constitute most of the adult U.S. population >50 years of age (60% of those 75 years of age and 70% of those 85 years of age).

The immune system does not function as well with advancing age (*5*). For example, T-cell functions diminish with age, as evidenced by the increased prevalence of anergy to mycobacterial and fungal skin-test antigens and the increased frequency and severity of herpes zoster infection with age. B-cell function diminishes, as seen with the lessened humoral response (immunoglobulin [Ig] M, IgG, and IgA) to certain vaccines (e.g., hepatitis B, influenza, pneumococcal vaccine), and the protective efficacy of these vaccines also decreases as recipients age (*6**,**7*).

## Differences in Immunologic Response by Sex

Very little is known about differences in the immunologic response to vaccines or their protective efficacy, according to sex and age. Higher antibody responses have been noted in women after hepatitis B vaccination (*8**,**9*). Trials are being completed to evaluate the effect of high dose varicella vaccine in reducing the high rates of herpes zoster and postherpetic neuralgia (*10**,**11*) in elderly. Some studies suggest that these problems preferentially involve older women. However, none of the differences reported between sexes are of a magnitude that affects any of the current vaccine recommendations.

The 2003–2004 Recommended Adult Immunization Schedule by Age Group, United States (*12*) (available from http://www.cdc.gov/nip/recs/adult-schedule.htm) covers the vaccines most commonly used for specific age brackets (Figure). We discuss the vaccines universally recommended for adults >50 years of age and selected vaccines for international travelers. Vaccine recommendations for special medical conditions (e.g., asplenia, pregnancy, diabetes, immunodeficiency, HIV infection, hepatitis exposures) may be found elsewhere (*12*).

**Figure:**
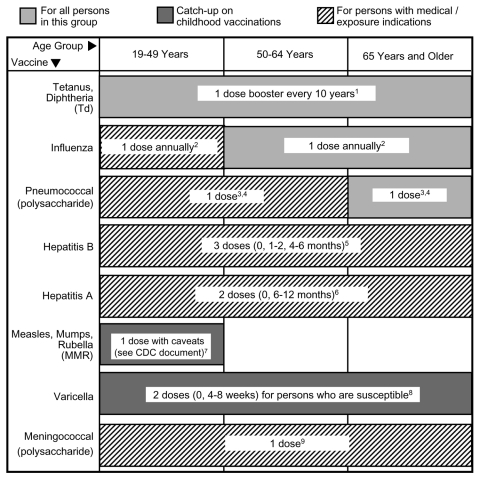
Recommended Adult Immunization Schedule, United States, 2003–2004 (*12*). This schedule indicates the recommended age groups for routine administration of currently licensed vaccines for persons >19 years of age. See http://www.cdc.gov/nip/recs/adult-schedule.htm for complete documentation of the numbered footnotes.

## Universal Vaccines for Persons >50 Years of Age

### Influenza

In an average year in the United States, influenza causes ≈36,000 deaths, 114,000 hospitalizations, 25 million physician visits, and an additional 30–60 million milder infections (*1*). Death and severity of illness are correlated with increasing age and underlying conditions. Persons at high risk for influenza complications include persons >65 years of age, residents of chronic-care facilities, and persons with chronic medical conditions, such as pulmonary, metabolic, or cardiovascular disorders; renal dysfunction; immunocompromised conditions; and splenic absence or dysfunction. Because ≈30% of the U.S. population 50–64 years of age have one or more conditions that warrant influenza vaccination (*1*) and because age-based recommendations are easier to implement, 50 years of age has now been established as the time for beginning the universal annual influenza vaccination (*13*).

The only influenza vaccine currently licensed for persons >50 years of age is the killed trivalent influenza vaccine (TIV), which is annually constituted to contain the two type A strains and one type B strain thought most likely to circulate in the next influenza season. The vaccine usually becomes available in late September and should be administered annually, ideally from September to November. If the circulating and vaccine strains are well matched, 70%–90% of healthy recipients <65 years of age will be protected against influenza. In elderly and immunocompromised recipients, disease prevention rates are lower because of decreased immune response, but the vaccine is still effective in reducing the severity of illness. In elderly persons living in nursing homes, influenza vaccine can be 50%–60% effective in preventing hospitalization and pneumonia and 80% effective in preventing influenza-related deaths (*14**–**16*). Substantial reductions of cardiac events and cerebrovascular disease, as well as pneumonia, among influenza vaccine recipients >65 years of age have been reported from a large study in Minnesota (*17*). If this finding is confirmed, it would be an added benefit of influenza vaccination in elderly populations.

Current rates of influenza vaccination by population group are shown in the Table (*1*). Although the rates in nursing home residents (83%) are approaching the goal (90%) set by the Healthy People 2010 initiative, the overall rates for the elderly have been stalled at 65% to 67% for the past 3 years and less that one third of persons at high risk in younger age groups have been vaccinated. Fully implementing the current influenza vaccine recommendations would prevent many illnesses and deaths annually, particularly in the elderly, and is a potentially cost-saving public health challenge.

**Table:** Influenza vaccination rates by population group^a^

Population group	Vaccinated (%)	By 2010 (%)^b^
Persons >65 y	67^c^	90
Nursing home patient	83	90
Persons at high-risk 18–64 y	29	60
Healthcare workers	36	60

^a^Centers for Disease Control and Prevention. MMWR Morb Mortal Wkly Rep. 2003;52(RR-8). ^b^Healthy People 2010 goals. ^c^Vaccination rates for all components of the over-65 age group in 2001 to 2002 were 70% for non-Hispanic whites, 47% for Hispanics, and 52% for non-Hispanic blacks.

Influenza vaccination of healthcare workers has been a long-standing recommendation for prevention of nosocomial spread of influenza, especially to persons at high risk. Vaccinating healthcare workers in nursing homes has been reported to reduce disease among the residents (*18**,**19*); therefore, the overall rate (36%) of vaccination among healthcare workers needs to be increased.

A trivalent, live, attenuated, cold-adapted influenza vaccine (LAIV-T) has been licensed for use in persons 5–49 years of age (*1**,**20*). The vaccine is administered by intranasal spray and induces local mucosal immunity as well as systemic immunity. This vaccine appears to provide protection similar to the TIV vaccine and may be superior in years when the vaccine strains do not closely match the circulating virus. LAIV-T requires special cold storage and is more expensive than TIV. At the time of licensure, not enough data regarding its use in elderly persons were presented, and approval for adult use was granted only up to 50 years of age. Studies in older adults are in progress.

The addition of neuraminidase inhibitors (oseltamivir and zanamivir) to the arsenal of chemoprophylactic and therapeutic drugs for influenza poses further options, especially for the elderly, who generally respond less well to vaccines. While previously available drugs, amantadine and rimantadine, have similar efficacy against influenza A and are less expensive, the newer drugs have fewer serious side effects and provide protection against influenza B as well. Data are limited and inconclusive concerning the effectiveness of these drugs for preventing or treating serious complications of influenza.

Questions remain about how best to prevent or modify influenza in the elderly. Should a vaccine with increased antigen dosage or different adjuvants be developed in the hope of improving the antibody levels in older persons? Would a vaccination schedule of twice per season, e.g., October/November and January/February, give more sustained protection to the elderly? Because the immunologic response diminishes with age, should those >80 years of age also receive antiviral chemoprophylaxis during influenza outbreaks? Should antiinfluenza drugs be added to the empiric initial treatment of patients hospitalized for community-acquired pneumonia during the influenza season?

### Pneumococcal Disease

The incidence of and deaths from invasive pneumococcal disease rise sharply among adults after 50 years of age (*3*). Invasive pneumococcal diseases, mainly bacteremia and meningitis, cause ≈9,000 deaths per year in the United States, with case-fatality rates that exceed 50% in the elderly. Estimates of pneumococcal pneumonia vary from 500,000 cases per year (*3**,**21*) to 106,000–175,000 cases per year (*22*), with case-fatality rates of 5% to 7% among the hospitalized elderly. Pneumococcal disease rates are substantially elevated in certain minority populations, including African-Americans, Alaska natives, and Native Americans.

The pneumococcal polysaccharide vaccine (PPV), which is available for use in adults, consists of purified capsular polysaccharide from the 23 serotypes of pneumococcus, which account for approximately 85%-90% of cases of invasive pneumococcal disease. Overall, this vaccine is ≈60% effective in preventing invasive pneumococcal disease caused by serotypes included in the vaccine but is less effective in the elderly (*23*). The vaccine has not demonstrated consistent benefit in preventing pneumococcal pneumonia (*23**–**25*).

The PPV vaccine is recommended for all adults >65 years (*12*) and for younger adults with chronic medical conditions, including diabetes, heart disease, chronic pulmonary disease (excluding asthma), chronic liver disease (including alcoholism), renal disease, asplenia or splenic dysfunction, HIV infection, and immunodeficiency states (*12*).

Recent surveys indicate that 66% of persons >65 years of age have received one or more PPV vaccinations (*26*). However, vaccination rates are lower for African-Americans and other minority groups and for younger adults with medical conditions that place them at higher risk for invasive pneumococcal disease. This situation indicates a major preventive medicine opportunity through full implementation of the current recommendations for PPV use. The antibody levels and protective efficacy provided by PPV vaccination gradually wane. Because PPV stimulates B-cells but does not induce T-cell memory, revaccination does not produce an anamnestic response. However, it usually elicits a rise in antibody that approximates the response to the initial vaccination (*27*).

In the absence of studies of the protective efficacy after revaccination and insufficient safety studies by age, advisory committees have been reluctant to issue firm directives. To date, routine revaccination is not recommended for immunocompetent persons who were first vaccinated >65 years of age. However, for persons who were first vaccinated before 65 years of age for reason of increased risk, a single revaccination is recommended (*12*). In the absence of data, some practicing physicians, given the overall good safety profile and low cost of PPV, have chosen to revaccinate elderly patients at 5- to 10-year intervals as a prudent response to the increasing rates of pneumococcal disease and deaths with age.

Additional questions about PPV recommendations exist. Should the vaccine indications be broadened to include those who smoke cigarettes, now shown to be a major risk factor for invasive pneumococcal disease (*28*), and minority groups who have 2–10 times more invasive pneumococcal disease than that of the general population? Should the age for universal vaccination of adults be lowered to 50 years of age (*29**,**30*) on the basis of the observations that ≈30% of adults 50–64 years of age have risk factors for which PPV is indicated (*13**,**29*)? Approximately 18% of the U.S population is made up of minority groups who have high rates of pneumococcal disease, and approximately one quarter of Americans 50–64 years of age smoke cigarettes. An added advantage to lowering the age of universal PPV vaccination to 50 years of age would be the "harmonization" of the adult pneumococcal vaccination schedule with the recommendations for influenza vaccine (*30*), thereby simplifying the system and hopefully improving the poor implementation rates of condition-based recommendations.

Finally, the development of a seven-valent pneumococcal conjugate vaccine for infants and young children has been a major advance that provides >90% protection against invasive pneumococcal disease in young children. Since young children are an important reservoir of pneumococcal carriage and spread it to others, vaccinating young children has provided a beneficial herd immunity that results in substantial reductions of pneumococcal disease in other children and adults (*31*). Developing a pneumococcal conjugate vaccine suitable for adults has been difficult.

### Tetanus-Diphtheria (Td) Toxoid Boosters

Tetanus toxoid and diphtheria toxoid are excellent immunogens. The primary vaccination series in childhood provides high-level protection and induces long-lasting immunologic memory, as evidenced by an anamnestic antibody response to Td after intervals of >30 years. High levels of primary vaccination and appropriate wound care (including Td boosters) are the cornerstones of tetanus prevention in the United States. The current recommendation that all adults in the United States receive Td boosters every 10 years has been poorly implemented, as evidenced by serosurveys showing that most adults >50 years of age lack protective levels of antibodies to either tetanus toxoid, diphtheria toxoid, or both (*32*). Despite this high level of serosusceptibility, tetanus (≈30 cases/y) and diphtheria (0–3 cases/y) are rare diseases and almost always occur in persons who never completed the full schedule of childhood vaccinations. Cost and benefit studies favor a policy of a single mid-life Td booster for persons who have completed the full pediatric series (*33*), and several advisory groups have recommended a booster at 50 years of age as an alternative to the current standard of decennial boosters (*12**,**34*). Consideration of reducing the frequency of Td boosters will be complicated by the proposed addition of the acellular pertussis vaccine to the adult Td formulation, as it is more costly and induces a shorter duration of antibody response than Td.

### Vaccines for Travelers >50 Years of Age

The increasing participation of older people in international travel raises an additional set of vaccination issues. Influenza and pneumococcal vaccinations should not be overlooked for travelers, especially during the different winter season in the Southern Hemisphere. Similarly, the crowding of people from many parts of the world on cruise ships or other international gatherings, is a setting in which influenza and other seasonal viruses may occur out of season. Hepatitis A vaccine is indicated for all travelers to areas of the world where sanitation or water safety are in question. Although approximately one half the U.S. population >50 years of age has serologic evidence of immunity to hepatitis A because of previous hepatitis A exposure, the pragmatic policy is to give hepatitis A vaccine rather than to serologically screen and vaccinate only the immunosusceptible persons. The vaccine is safe and highly protective when given preexposure and may have benefit when administered early in the postexposure setting. Two doses spaced >6 months apart are recommended, although serologic and epidemiologic data suggest that one dose may provide long-term protection. No data are available to indicate the duration of protection in the elderly. For persons who also require hepatitis B vaccination, a combined hepatitis A- hepatitis B vaccine (Twinrix) provides a convenient three-dose method of vaccination. Hepatitis B vaccine is indicated for persons planning long-term travel (generally >6 weeks) to areas of high hepatitis B prevalence or who anticipate parenteral exposure (dental procedures, needle exposure, blood products) or sexual exposure to hepatitis B virus. The standard three-dose schedule is 0, 1, and 6 months, although an accelerated schedule of 0, 1, and 4 weeks offers good short-term protection but requires a fourth dose at 1 year. The duration of protection is not well defined but appears to exceed the duration of the antibody response; therefore, no firm recommendation exists regarding booster doses. However, the immune response to hepatitis B vaccine diminishes sharply with age and for older persons. For older persons with continual or repeated exposures to hepatitis B, measuring antibody levels and considering boosters for those with low levels are advisable. Yellow fever vaccine, a live, attenuated viral vaccine, is indicated for travelers to disease-endemic areas of South America and sub-Saharan Africa, and several countries require proof of vaccination as a condition of entry. Cases of yellow fever among U.S. travelers have been few but have increased in the last decade, and some deaths have occurred. On the other hand, recent reports have described the rare occurrence of a systemic illness mimicking yellow fever after yellow fever vaccine administration to elderly persons (*35*). Therefore, the vaccine should not be administered to persons (especially the elderly) who are not traveling to yellow fever–endemic areas. Recommendations for the other travel related vaccines (meningococcal, typhoid fever, polio, rabies, and Japanese encephalitis) are the same for all adult age groups and are fully described in Health Information for International Travelers (*36*).

## Future Developments

Because increasing age is associated with increasing rates of herpes zoster and post-herpetic neuralgia, studies are under way to evaluate the administration of high-dose varicella vaccine to persons >60 years of age in an effort to boost antivaricella antibodies and reduce the late complications of varicella. Several promising vaccines important to women are in development to prevent cervical cancer (human papillomavirus vaccine), sexually transmitted diseases, and transmission of pathogens (e.g., group B streptococcus, cytomegalovirus) from pregnant women to their newborns, but these vaccines are intended for younger populations and will not be important vaccines for persons >50 years of age. The generic problems of lessened vaccine response and efficacy with advanced age call for increased research regarding the immune response in the elderly. Approaches that appear promising include developing age- and sex-specific adjuvants, considering different antigen doses and vaccination schedules that offer the possibility of improving the immunologic response, and vaccines to boost T-cell and phagocytic host defenses. Advances in genetics will facilitate the identification of subpopulations with unique vaccine responses. Also, the ability to genetically engineer vaccines with higher antigen concentrations and the capacity to combine a variety of antigens offer the promise of broader protection with simplified vaccine schedules.

## Conclusion

Preventing illnesses and deaths in older populations from diseases that are preventable through vaccines is a leading public health challenge. Our understanding of the effects of age and sex on the immune system is limited. Fully implementing vaccine recommendations, particularly for influenza and pneumococcal vaccines, offers the immediate prospect of saving thousands of lives and reducing major illnesses among persons >50 years of age.
